# Chromatin remodeling controls Kaposi's sarcoma-associated herpesvirus reactivation from latency

**DOI:** 10.1371/journal.ppat.1007267

**Published:** 2018-09-13

**Authors:** Sharon E. Hopcraft, Samantha G. Pattenden, Lindsey I. James, Stephen Frye, Dirk P. Dittmer, Blossom Damania

**Affiliations:** 1 Lineberger Comprehensive Cancer Center, Department of Microbiology and Immunology, University of North Carolina at Chapel Hill, Chapel Hill, NC, United States of America; 2 Center for Integrative Chemical Biology and Drug Discovery, Division of Chemical Biology and Medicinal Chemistry, Eshelman School of Pharmacy, University of North Carolina at Chapel Hill, Chapel Hill, NC, United States of America; University of Washington, UNITED STATES

## Abstract

Kaposi’s sarcoma-associated herpesvirus (KSHV) is the etiologic agent of three human malignancies, the endothelial cell cancer Kaposi’s sarcoma, and two B cell cancers, Primary Effusion Lymphoma and multicentric Castleman’s disease. KSHV has latent and lytic phases of the viral life cycle, and while both contribute to viral pathogenesis, lytic proteins contribute to KSHV-mediated oncogenesis. Reactivation from latency is driven by the KSHV lytic gene transactivator RTA, and RTA transcription is controlled by epigenetic modifications. To identify host chromatin-modifying proteins that are involved in the latent to lytic transition, we screened a panel of inhibitors that target epigenetic regulatory proteins for their ability to stimulate KSHV reactivation. We found several novel regulators of viral reactivation: an inhibitor of Bmi1, PTC-209, two additional histone deacetylase inhibitors, Romidepsin and Panobinostat, and the bromodomain inhibitor (+)-JQ1. All of these compounds stimulate lytic gene expression, viral genome replication, and release of infectious virions. Treatment with Romidepsin, Panobinostat, and PTC-209 induces histone modifications at the RTA promoter, and results in nucleosome depletion at this locus. Finally, silencing Bmi1 induces KSHV reactivation, indicating that Bmi1, a member of the Polycomb repressive complex 1, is critical for maintaining KSHV latency.

## Author summary

Kaposi’s sarcoma-associated herpesvirus (KSHV) is the causative agent of three human malignancies. KSHV establishes latent infection but can be reactivated from latency to the lytic phase of the viral life cycle to propagate. KSHV reactivation from latency is controlled by epigenetic changes at the promoter of the lytic gene transactivator, RTA. Here a novel inhibitor of a member of Polycomb repressive complex 1, Bmi1, stimulates KSHV reactivation from latency by altering histone modifications and nucleosome density at the RTA promoter. We also identify three additional inhibitors of histone deacetylases and bromodomain-containing proteins that induce KSHV reactivation from latency.

## Introduction

Kaposi’s sarcoma-associated herpesvirus (KSHV), also known as human herpesvirus 8, is the causative agent of three human malignancies, the endothelial cell cancer Kaposi’s sarcoma (KS), as well as two B cell cancers, primary effusion lymphoma (PEL) and multicentric Castleman’s disease (reviewed in [1]). KSHV establishes latent infections, where only a few viral genes and microRNAs are expressed, but can be reactivated from latency to the lytic phase of the viral life cycle, where all viral genes are expressed, the viral genome is replicated, and progeny virions are released. Although the majority of KSHV positive cells in PEL and KS are latently infected, the virus undergoes spontaneous reactivation in a fraction of these cells [2]. It is thought that spontaneous reactivation contributes to KSHV maintenance, and that certain lytic proteins shape the clinical pathology of KS [3, 4].

Upon initial infection, the KSHV genome is rapidly chromatinized [5, 6]. Epigenetic modifications play a role in KSHV reactivation from latency as histone deacetylase (HDAC) inhibitors such as sodium butyrate (NaB) and DNA demethylating agents such as 5-azacytidine stimulate reactivation *in vitro* [7–9] and in patients [10]. Expression of the KSHV lytic gene transactivator RTA is necessary and sufficient to drive the lytic program [11–14]. During latency, the RTA promoter is associated with HDACs and with Enhancer of Zeste Homolog 2 (EZH2) [5, 15–17]. EZH2 is the catalytic subunit of Polycomb Repressive Complex 2 (PRC2), which trimethylates histone 3 on lysine residue 27 (H3K27me3). Thus, histones at the RTA promoter are hypoacetylated and bear H3K27me3, both of which are associated with repressed transcription. The KSHV latency-associated nuclear antigen (LANA/ORF73) also antagonizes the RTA promoter [18] and interacts with bromodomain-containing proteins [19–21], which bind acetylated histones. Analysis of nucleosome density by formaldehyde-assisted isolation of regulatory elements (FAIRE) did not reveal regions of open chromatin in the RTA promoter in latent KSHV genomes [22]. On the other hand, markers of active transcription, like histone 3 lysine 4 trimethylation (H3K4me3), are also present at this locus, indicating that this region is transcriptionally repressed but is poised for rapid induction of RTA expression [5, 16]. During reactivation induced by treatment with NaB, there is increased histone acetylation, decreased association with EZH2, and decreased levels of H3K27me3 at the RTA promoter [16]. Furthermore, the Ini1/SNF5 chromatin remodeling complex is recruited to this locus, and nucleosomes are rapidly depleted as demonstrated by restriction endonuclease accessibility and micrococcal nuclease assays [15]. Thus, RTA transcription and KSHV reactivation are particularly sensitive to epigenetic changes occurring within the KSHV genome.

We previously conducted screens for cellular kinases and natural product extracts that repress or activate KSHV reactivation, respectively, which provided proof of principle that KSHV reactivation screens are possible and represent a means to identify novel regulators of RTA transcription [23, 24]. To gain further insight into epigenetic control of the latent to lytic switch, we screened a library of known small molecule inhibitors that target epigenetic regulatory proteins such as histone writers, readers, and erasers for their ability to stimulate KSHV reactivation. Histone writers add post-translational modifications to histones, and include histone methyltransferases like EZH2, as well as histone acetyltransferases, among many others. Histone erasers, such as HDACs and histone demethylases, remove modifications from histones. Histone readers bind to histone modifications and impact a variety of processes like chromatin remodeling and transcription. These proteins include methyl-lysine readers, such as chromodomain-containing proteins, and acetyl-lysine readers, the most common of which are the bromodomain-containing proteins. Thus, by screening inhibitors that target a range of chromatin regulatory proteins, we aimed to identify host proteins that are critical for the maintenance of KSHV latency.

## Results

### Screen for inhibitors that induce KSHV reactivation from latency

**Fig 1:**
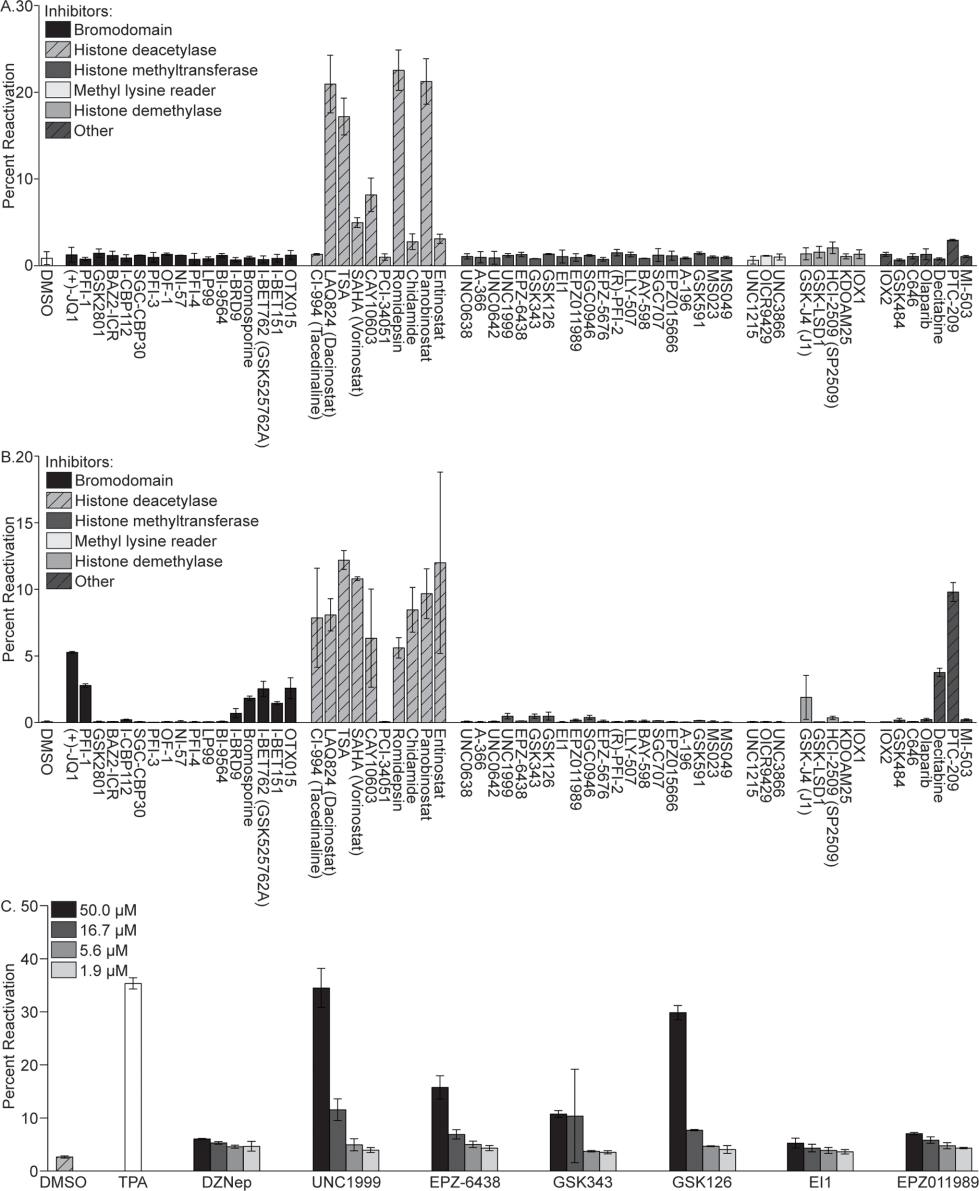
Inhibitor screen for epigenetic regulatory proteins that induce KSHV reactivation from latency. BJAB-KSHV.219 cells were incubated with 1 μM of each compound for 48 hours in (A) or 10 μM of each compound for 120 hours in (B), and the percentage of GFP positive cells expressing RFP (percent reactivation) was determined by FACS analysis. (C) BJAB-KSHV.219 cells were incubated with EZH2 inhibitors at concentrations of 50, 16.7, 5.6, and 1.9 μM for 120 hours. Percent reactivation was determined by FACS analysis. Data are representative of two independent experiments performed in duplicate; mean and standard error are shown.

To identify host chromatin-modifying proteins that are essential for maintaining KSHV latency, we screened 62 known small molecule inhibitors that target epigenetic regulatory proteins for their ability to stimulate KSHV reactivation. These compounds fell within several categories, including inhibitors of bromodomains, HDACs, histone methyltransferases, methyl-lysine readers, histone demethylases, and others. Each compound, or DMSO as a negative control, was incubated at a concentration of 1 μM for 48 hours with BJAB cells latently infected with rKSHV.219, a recombinant virus that expresses GFP from the constitutively active EF-1α promoter, expresses RFP from the KSHV PAN lytic promoter, and expresses a puromycin resistance gene as a selectable marker [25]. BJAB-KSHV.219 cells were selected for this study as regions of open chromatin in the KSHV genome are conserved in these cells and PEL cells [22]. Following inhibitor treatment, GFP and RFP expression were assayed by flow cytometry. The compounds that induced appreciable KSHV reactivation after 48 hours were the HDAC inhibitors LAQ824 (Dacinostat), Trichostatin A (TSA), SAHA (Vorinostat), CAY10603, Romidepsin, and Panobinostat, where 5–25% of treated cells were RFP positive (Fig 1A). The same compounds induced reactivation in latently infected 293 cells (S1A Fig). The TSA and SAHA results are consistent with previously published reports where these compounds induced KSHV reactivation in a subset of latently infected cells [15, 26, 27], thus validating the screen. Although it is well established that HDAC inhibitors can induce KSHV reactivation from latency, two of the most potent stimulators of reactivation, the pan-HDAC inhibitors Romidepsin and Panobinostat, have not previously been shown to stimulate KSHV reactivation.

To identify additional inhibitors that reactivate KSHV from latency, several modifications were made to the experimental design. First, we increased the working concentration of each compound to 10 μM. Second, as it can take several days for an appreciable decrease in histone methylation to occur following inhibition of methyltransferase activity [28], we increased the time of incubation with each compound to five days. Under these conditions, one of the most effective stimulators of KSHV reactivation from latency was PTC-209 [29], where reactivation occurred in approximately 10% of treated cells (Fig 1B). PTC-209 is reported to reduce transcription of B cell-specific Moloney murine leukemia virus integration site 1 (Bmi1), although the molecular details of its mechanism of action and selectivity versus other potential targets has not been well defined. Bmi1 is a member of Polycomb Repressive Complex 1 (PRC1), which monoubiquitinates histone 2A on lysine residue 119 (H2AK119ub) [30]. Bmi1 functions to enhance the enzymatic activity of the PRC1 catalytic subunit, a RING1 ubiquitin ligase [31]. Additionally, all of the HDAC inhibitors except PCI-34051 induced reactivation in 5–12% of treated cells. Several bromodomain inhibitors also stimulated reactivation, all of which target the BET family of bromodomains which includes BRD2, -3, and -4. The most potent of these inhibitors was (+)-JQ1, where reactivation occurred in 5% of treated cells. Other bromodomain inhibitors that induced reactivation, including PFI-1, Bromosporine, I-BET762 (GSK525762A), I-BET151, and OTX015, were less effective, and stimulated reactivation in 2–3% of treated cells. KSHV reactivation was also induced by the JMJD3 histone demethylase inhibitor GSK-J4 and the DNA methyltransferase inhibitor Decitabine in 2% and 4% of treated cells, respectively. The same set of compounds also induced KSHV reactivation in 293-KSHV.219 cells (S1B Fig), although PTC-209 was somewhat less effective in these cells, where reactivation was induced in 3.3% of treated cells. As PTC-209 and (+)-JQ1 were the most potent of the non-HDAC inhibitors, these were also selected for further analysis.

Under the culture conditions tested thus far, the EZH2 inhibitors included in the screen, UNC1999, EPZ-6438, GSK343, GSK126, EI1, and EPZ011989, did not induce appreciable KSHV reactivation. This was surprising given that H3K27me3 is associated with latent KSHV genomes and suppressed RTA expression, and the EZH2 inhibitor 3-Deazaneplanocin A (DZNep) has previously been demonstrated to induce KSHV reactivation from latency [16]. To determine the effectiveness of these EZH2 inhibitors at higher concentrations, we tested 3-fold serial dilutions of each compound starting from 50 μM. UNC1999 and GSK126 were the most potent inducers of KSHV reactivation, which was stimulated in approximately 35 percent of cells treated with the highest concentration of these inhibitors (Fig 1C). This was comparable to the level of reactivation induced by the positive control, 12-O-tetradecanoyl-phorbol 13-acetate (TPA). EPZ-6438 and GSK343 induced reactivation in 16 and 11 percent of cells, respectively. DZNep, EI1, and EPZ011989 stimulated KSHV reactivation in 6, 5, and 7 percent of cells, respectively. Thus, our data is consistent with previously published reports describing a role for EZH2 in the maintenance of KSHV latency.

### Romidepsin, Panobinostat, (+)-JQ1, and PTC-209 induce KSHV lytic gene expression in PEL cells

**Fig 2:**
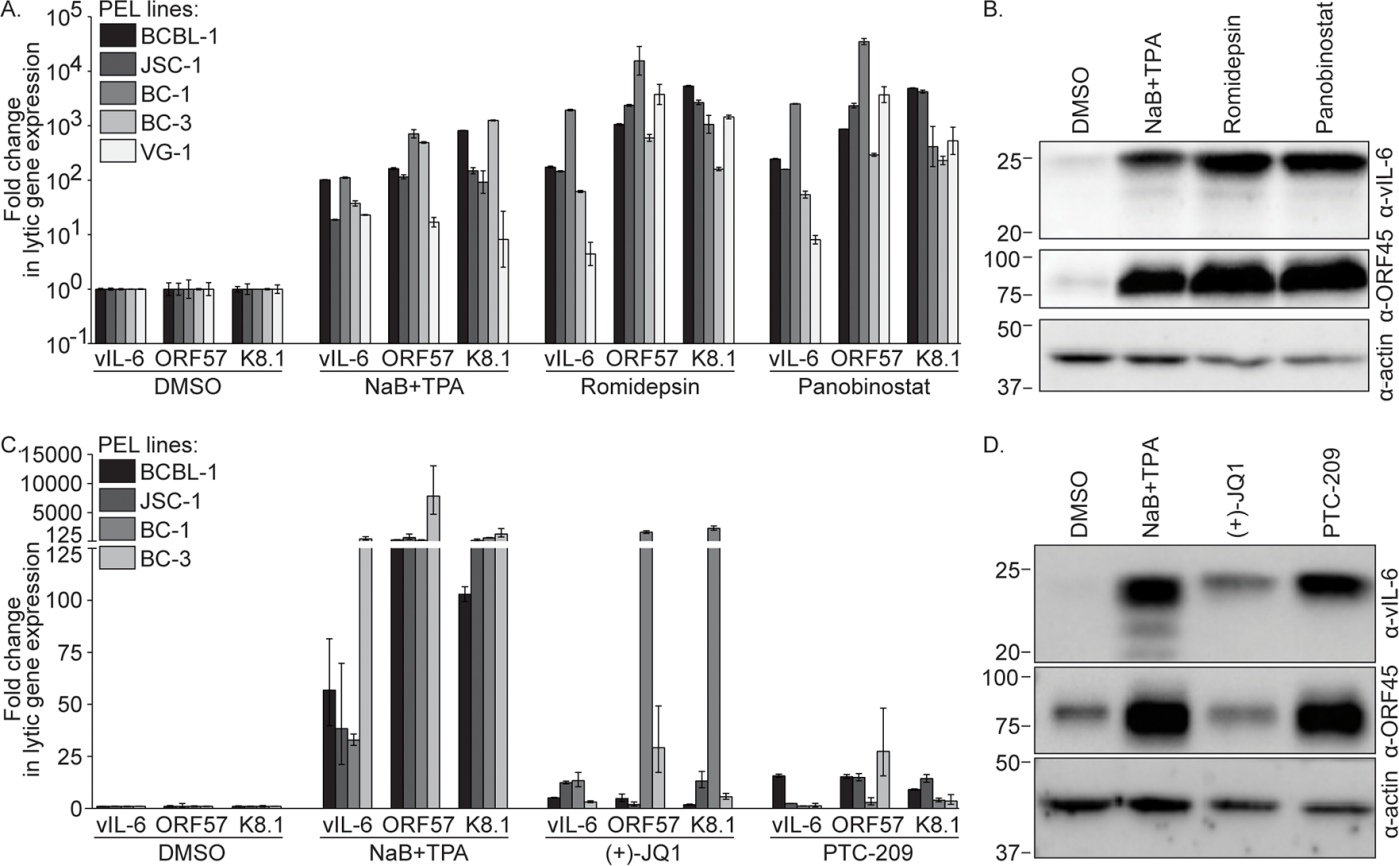
Romidepsin, Panobinostat, (+)-JQ1, and PTC-209 induce KSHV lytic gene expression. (A) RT-qPCR analysis for KSHV lytic transcript levels was performed on PEL cells treated with DMSO, NaB+TPA, or 1 μM Romidepsin or Panobinostat for 48 hours. Lytic transcript levels were normalized to GAPDH, and the fold difference between DMSO and compound treated cells was calculated. (B) Immunoblot analysis for vIL-6, ORF45, or actin was performed on lysates from BCBL-1 cells treated as in (A). Approximate molecular mass (kDa) marker positions are indicated to the left of each blot. (C) RT-qPCR analysis for KSHV lytic transcript levels was performed as in (A) on PEL cells treated with DMSO, NaB+TPA, or 10 μM (+)-JQ1 or PTC-209 for 120 hours. (D) Immunoblot analysis was performed on lysates from BCBL-1 cells treated as in (C). Data are representative of at least two independent experiments performed in triplicate; mean and standard error are shown.

To confirm that our compounds of interest, Romidepsin, Panobinostat, (+)-JQ1, and PTC-209, induce KSHV reactivation in PEL cells, various PEL lines were incubated with each compound, NaB+TPA as a positive control, or DMSO, and KSHV lytic transcript levels were analyzed by RT-qPCR. Romidepsin and Panobinostat induced expression of vIL-6, ORF57, and K8.1 in all PEL lines tested at levels similar to or exceeding those induced by NaB+TPA (Fig 2A). For example, NaB+TPA treatment induced lytic gene expression by approximately 100- to 1,000-fold in BCBL-1 cells compared to the DMSO control, while Romidepsin and Panobinostat induced lytic gene expression by approximately 200- to 5,000-fold in these cells. Furthermore, we observed increased vIL-6 and ORF45 protein levels in NaB+TPA, Romidepsin, and Panobinostat treated cells compared to the control (Fig 2B). Treatment with (+)-JQ1 and PTC-209 also induced KSHV lytic gene expression in all the PEL lines tested, albeit at more modest levels than those induced by NaB+TPA (Fig 2C). Expression of vIL-6, ORF57, and K8.1 was induced in NaB+TPA treated cells by levels ranging from 30- to 8000-fold compared to DMSO treated cells. In BCBL-1, JSC-1, and BC-3 cells, (+)-JQ1 induced expression of these genes to levels 2- to 30-fold higher than in DMSO treated cells. BC-1 cells were particularly responsive to (+)-JQ1 treatment, where ORF57 and K8.1 expression were induced to levels approximately 1000- to 2000-fold greater than in DMSO treated cells. Following treatment with PTC-209, vIL-6 expression increased in BCBL-1 and JSC-1 cells by approximately 16- and 2-fold, respectively, and ORF57 and K8.1 expression increased in all the PEL lines tested by 3- to 30-fold. We observed increased vIL-6 protein levels in (+)-JQ1 treated BCBL-1 cells compared to the control, and increased vIL-6 and ORF45 protein levels in PTC-209 treated cells (Fig 2D). Thus, Romidepsin, Panobinostat, (+)-JQ1, or PTC-209 treatment induces KSHV lytic gene expression in PEL cells.

**Fig 3:**
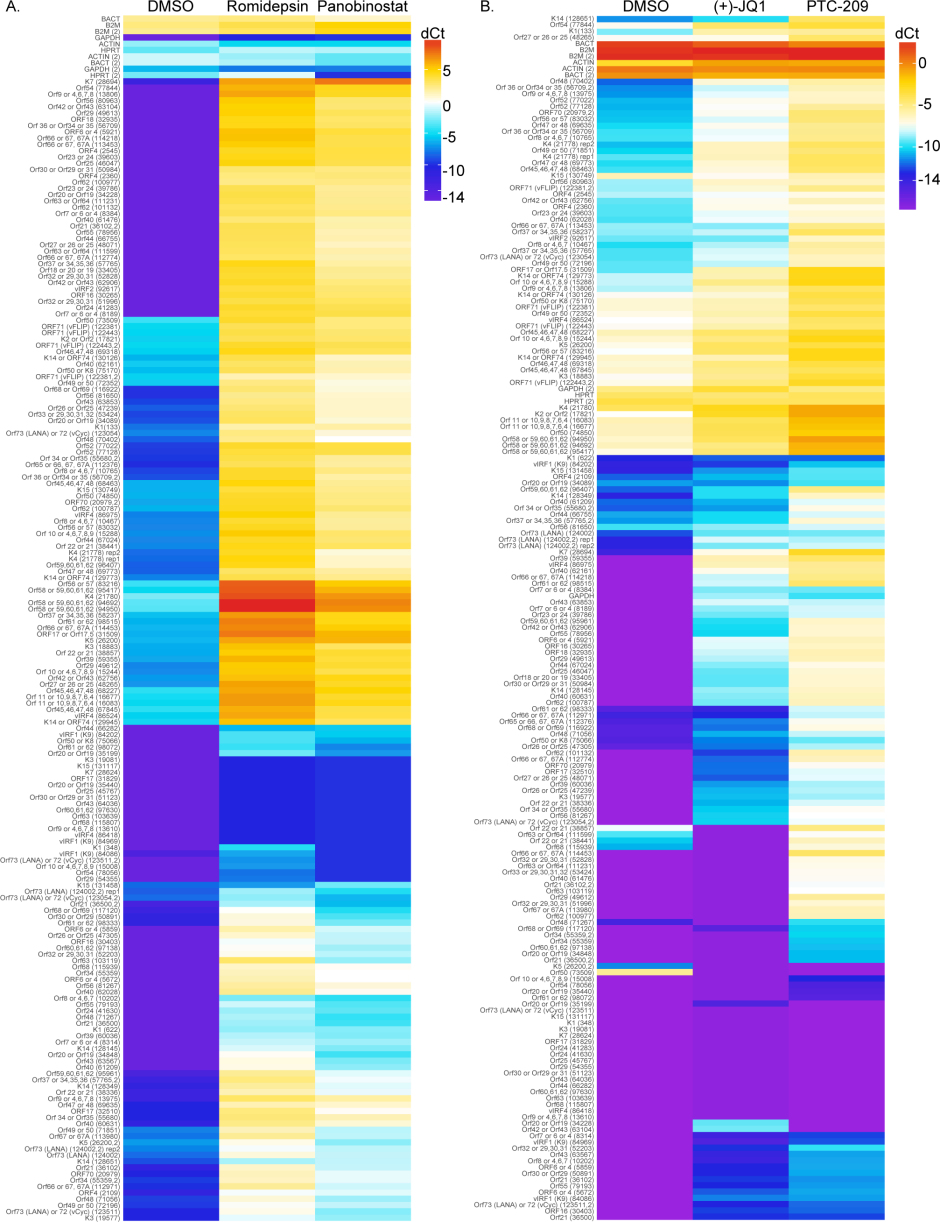
Romidepsin, Panobinostat, (+)-JQ1, and PTC-209 induce global KSHV gene expression. KSHV whole genome transcriptional profiling was performed on BCBL-1 cells treated with DMSO or 1 μM Romidepsin or Panobinostat for 48 hours (A), or with 10μM (+)-JQ1 or PTC-209 for 120 hours (B). mRNA levels of viral genes were normalized to the mRNA levels of multiple cellular housekeeping genes to yield dCt as a measure of relative expression. Higher transcript expression levels are indicated by red and lower expression levels by blue.

To test the impact of compound treatment on global KSHV gene expression, we performed KSHV whole genome transcriptional profiling on BCBL-1 cells incubated with DMSO as a negative control, Romidepsin or Panobinostat (Fig 3A), or (+)-JQ1 or PTC-209 (Fig 3B). Treatment with each compound induced broad KSHV gene expression compared to DMSO treated cells, confirming that Romidepsin, Panobinostat, (+)-JQ1, and PTC-209 stimulate KSHV lytic gene expression.

### Romidepsin, Panobinostat, and PTC-209 induce KSHV genome replication and virion production in PEL cells

**Fig 4:**
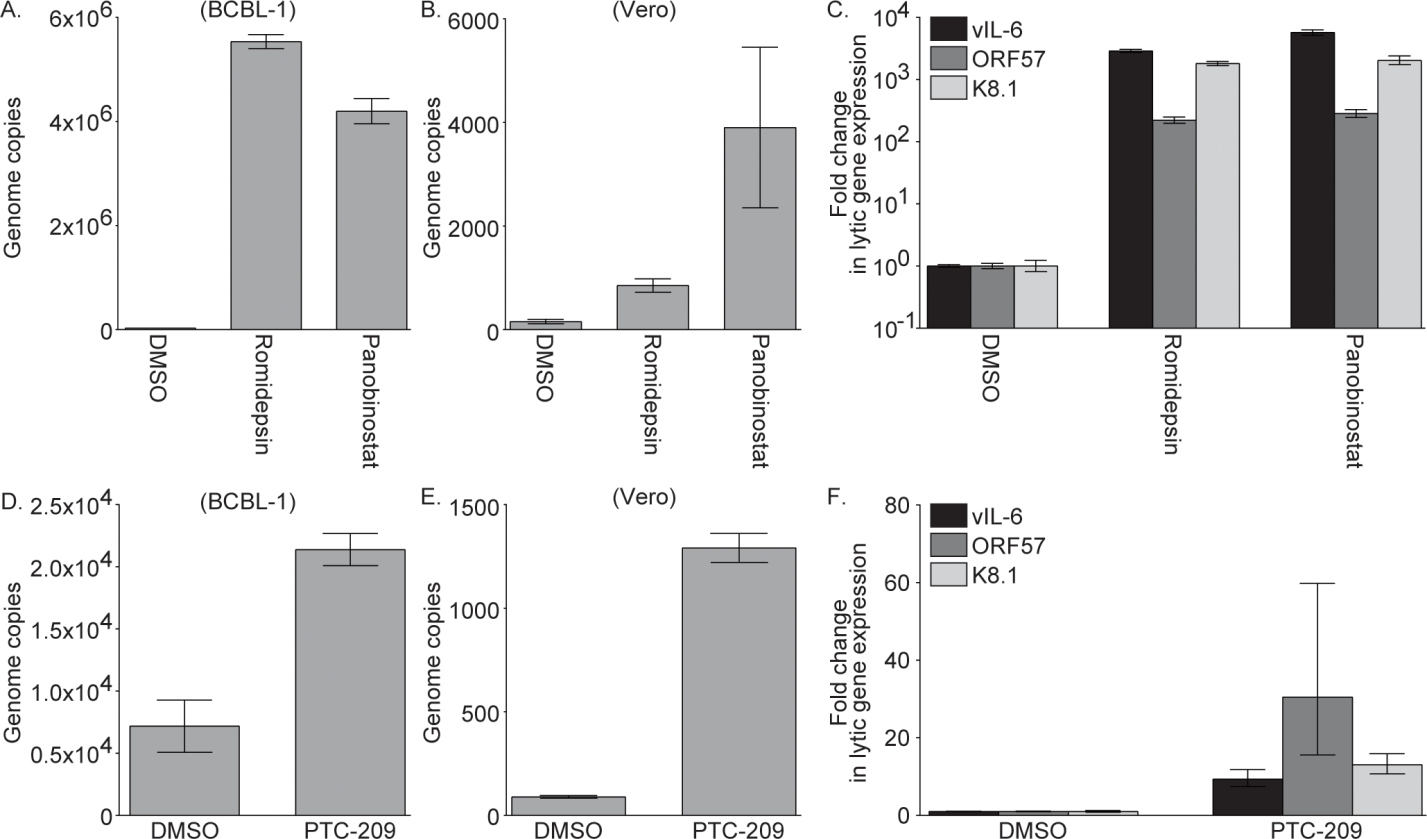
Romidepsin, Panobinostat, and PTC-209 induce KSHV genome replication and release of infectious virions. (A) qPCR analysis for cell-associated KSHV genome copies was performed on BCBL-1 cells treated with DMSO or 1 μM Romidepsin or Panobinostat for five days. Naïve Vero cells were infected with supernatants collected from BCBL-1 cells treated as in (A), and KSHV genome copies in the infected cells were determined by qPCR (B), and KSHV lytic transcript levels were assayed by RT-qPCR (C). Lytic transcript levels were normalized to GAPDH, and the fold difference between DMSO and compound treated cells was calculated. (D) qPCR analysis for cell-associated KSHV genome copies was performed on BCBL-1 cells treated with DMSO or 10 μM PTC-209 for seven days. Naïve Vero cells were infected with supernatants collected from BCBL-1 cells treated as in (D), and KSHV genome copies in the infected cells were determined by qPCR (E), and KSHV lytic transcript levels were assayed by RT-qPCR (F). Lytic transcript levels were normalized to GAPDH, and the fold difference between DMSO and PTC-209 treated cells was calculated.

As a thorough analysis of the mechanism by which (+)-JQ1 stimulates KSHV reactivation from latency has recently been published [32], we continued our studies with Romidepsin, Panobinostat, and PTC-209. To determine if these compounds induce KSHV genome replication, KSHV copy number in DMSO and inhibitor treated BCBL-1 cells was determined by qPCR. KSHV copy number was increased by at least 100-fold in Romidepsin and Panobinostat treated cells compared to DMSO treated cells, confirming that Romidepsin and Panobinostat induce KSHV genome replication (Fig 4A). To test the ability of inhibitor treated cells to produce infectious virions, supernatants from DMSO, Romidepsin, and Panobinstat treated cells were collected and used to infect naïve Vero cells, where KSHV copy number in the infected Vero cells was determined by qPCR. We found a 5- and 25-fold difference in KSHV copy number in Vero cells infected with supernatants collected from Romidepsin and Panobinostat treated versus DMSO treated BCBL-1 cells, respectively (Fig 4B). To probe viral gene expression in infected Vero cells, KSHV transcript levels were assayed by RT-qPCR. We found an approximately 200- to 5000-fold increase in levels of vIL-6, ORF57, and K8.1 transcripts in Vero cells infected with supernatants collected from Romidepsin or Panobinostat versus DMSO treated BCBL-1 cells (Fig 4C). Similarly, KSHV copy number was increased in PTC-209 treated BCBL-1 cells by 3-fold compared to DMSO treated cells (Fig 4D), and there was a 15-fold difference in KSHV copy number in Vero cells infected with supernatants collected from PTC-209 versus DMSO treated BCBL-1 cells (Fig 4E). vIL-6, ORF57, and K8.1 were expressed at levels 9- to 30-fold higher in Vero cells infected with supernatants collected from PTC-209 versus DMSO treated BCBL-1 cells (Fig 4F). Taken together, these data demonstrate that Romidepsin, Panobinostat, and PTC-209 induce complete KSHV reactivation from latency.

### Romidepsin, Panobinostat, and PTC-209 treatment induces histone modifications at the RTA promoter

**Fig 5:**
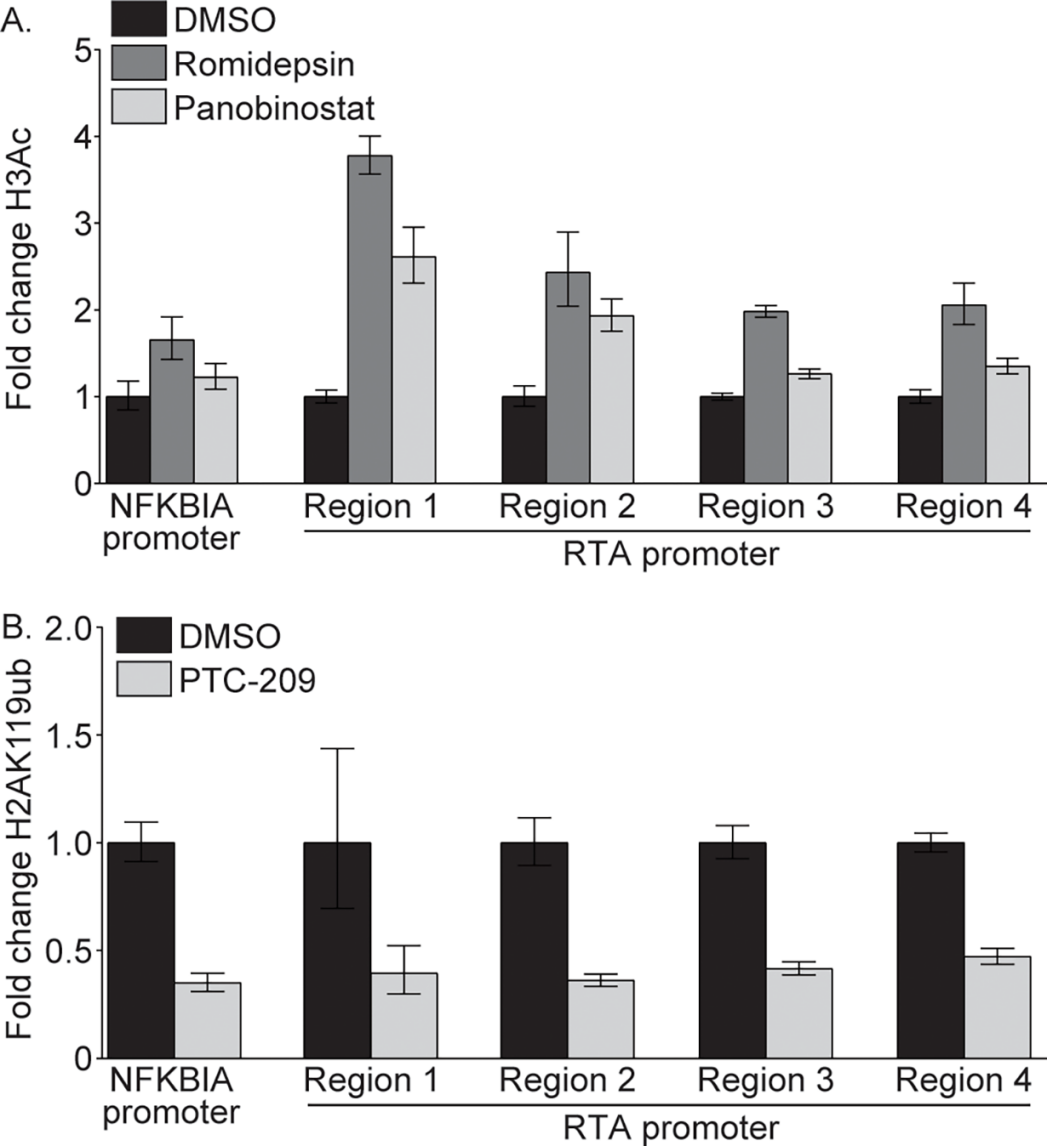
Romidepsin, Panobinostat, and PTC-209 impact histone modifications at the RTA promoter. (A) BCBL-1 cells were treated with DMSO or 1 μM Romidepsin or Panobinostat for four hours, and levels of H3Ac and total H3 at the RTA promoter were determined by ChIP-qPCR. Levels of H3Ac were normalized to total levels of H3, and the fold difference between DMSO and compound treated cells was calculated. (B) BCBL-1 cells were treated with DMSO or 10 μM PTC-209 for five days, and levels of H2AK119ub and total H2A at the RTA promoter were determined by ChIP-qPCR. Levels of H2AK119ub were normalized to total levels of H2A, and the fold difference between DMSO and PTC-209 treated cells was calculated. Data are representative of three independent experiments.

To probe the mechanism by which Romidepsin, Panobinostat, and PTC-209 stimulate KSHV reactivation, we performed chromatin immunoprecipitation (ChIP) on compound treated BCBL-1 cells. We hypothesized that Romidepsin and Panobinostat treatment would increase levels of acetylated histone 3 (H3Ac) at the RTA promoter, while PTC-209 treatment would reduce levels of H2AK119ub, which is associated with heterochromatin and repressed gene expression [30]. To test this hypothesis, we performed ChIP on DMSO, Romidepsin, or Panobinostat treated BCBL-1 cells using total H3, H3Ac, or control IgG antibodies. Following treatment with Romidepsin and Panobinostat, the amount of H3Ac increased at the promoter of the cellular gene NFKBIA, and at four regions of the RTA promoter by 1.3- to 3.8-fold compared to cells treated with DMSO (Fig 5A). Furthermore, ChIP-qPCR analysis following PTC-209 treatment using total H2A, H2AK119ub, or control IgG antibodies revealed a greater than 2-fold reduction in H2AK119ub at the NFKBIA promoter and across the RTA promoter in PTC-209 treated compared to DMSO treated cells (Fig 5B). These data confirm that treatment with Romidepsin, Panobinostat, and PTC-209 results in histone modifications at the RTA promoter that are favorable to RTA transcription.

### Romidepsin, Panobinostat, and PTC-209 treatment results in open chromatin at the RTA promoter

**Fig 6:**
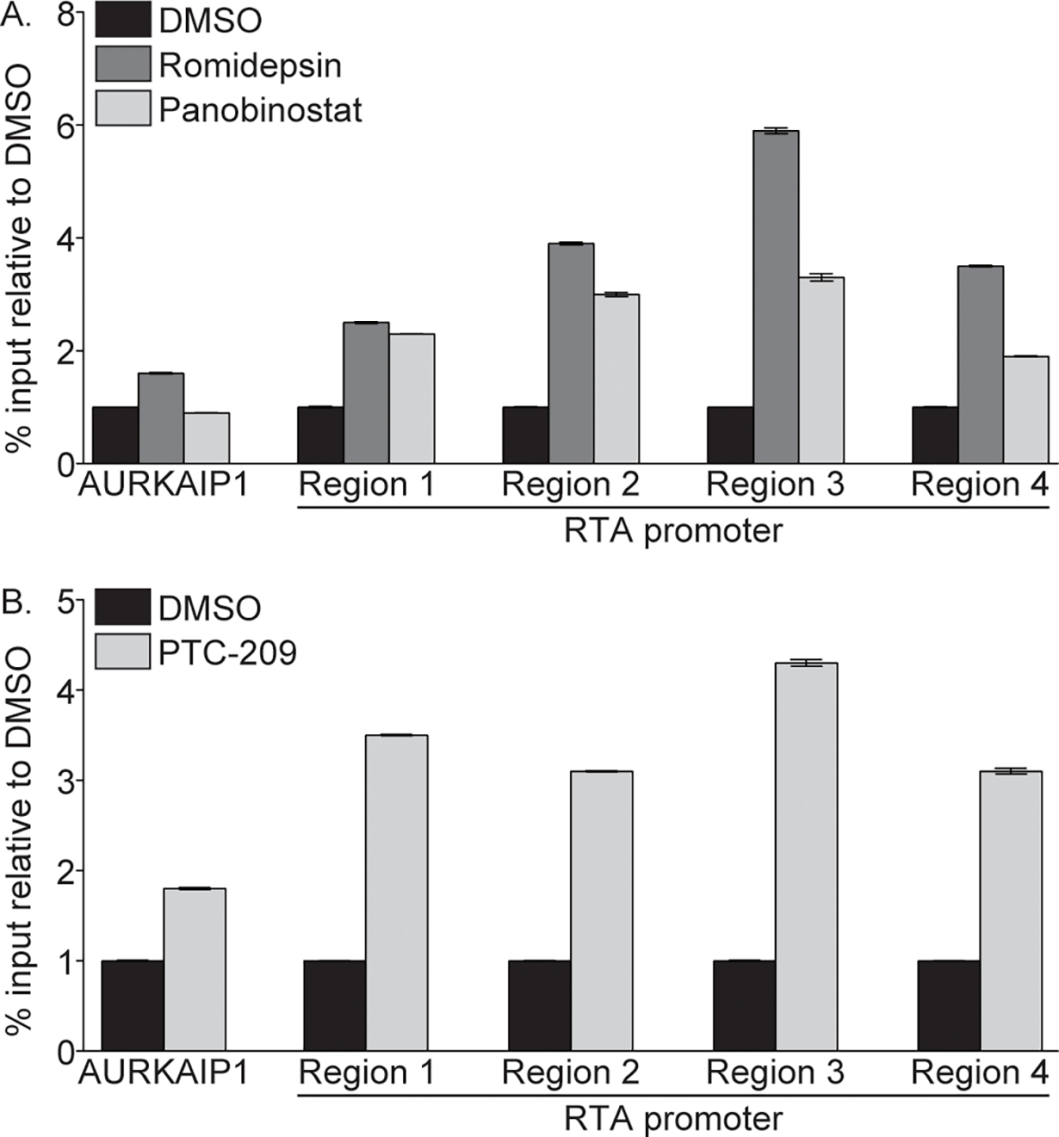
Romidepsin, Panobinostat, and PTC-209 induce nucleosome depletion at the RTA promoter. Nucleosome density was assayed by FAIRE-qPCR in 293-KSHV.219 cells treated with DMSO or 1 μM Romidepsin or Panobinostat (A), or DMSO or 10 μM PTC-209 (B). Data are representative of at least three independent experiments.

To determine if increased levels of H3Ac and decreased levels of H2AK119ub result in changes in chromatin accessibility at the RTA promoter, we probed nucleosome density in this region by FAIRE analysis. FAIRE relies on differential crosslinking efficiency between DNA bound by nucleosomes versus DNA in nucleosome-depleted regions, where an increase in FAIRE signal indicates a reduction in nucleosome density [33–35]. As a control, we probed the FAIRE signal at AURKAIP1, a region of host chromatin that is consistently nucleosome depleted [36]. Romidepsin and Panobinostat treatment resulted in a 1.9- to 5.9-fold increase in the FAIRE signal at four regions of the RTA promoter compared to DMSO treated cells (Fig 6A). Similarly, the FAIRE signal at the RTA promoter increased following PTC-209 treatment by 3.1- to 4.3-fold (Fig 6B). These data indicate that nucleosomes are depleted at the RTA promoter upon compound treatment.

### Treatment with Romidepsin and Panobinostat, but not PTC-209, alters chromatin accessibility across the KSHV genome

**Fig 7:**
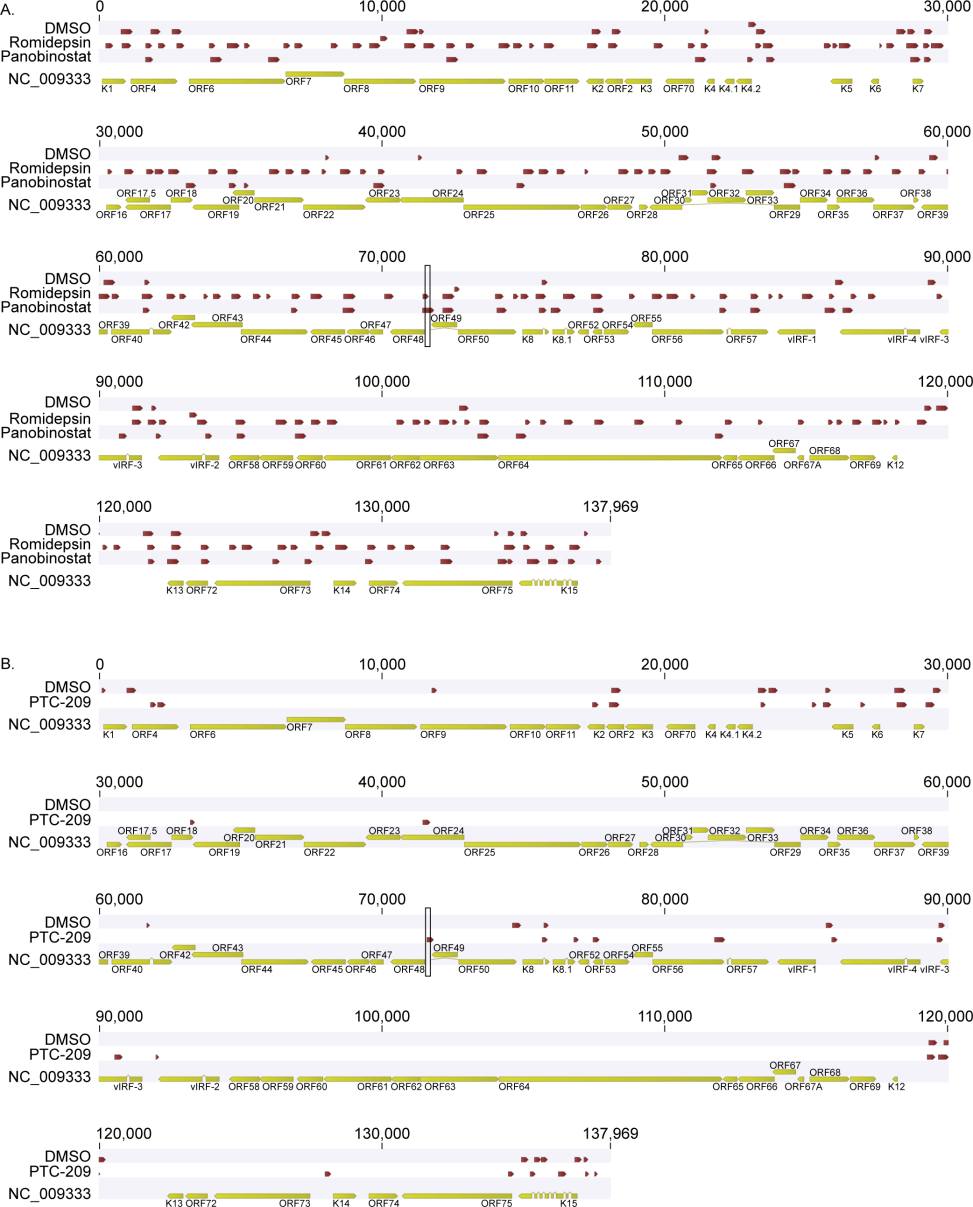
Romidepsin and Panobinostat, but not PTC-209, alter chromatin accessibility across the KSHV genome. Nucleosome density was assayed by FAIRE-seq in 293-KSHV.219 cells treated with DMSO or 1 μM Romidepsin or Panobinostat (A), or DMSO or 10 μM PTC-209 (B). Sequencing reads were mapped to the KSHV reference genome NC_009333, and mapped reads from biological replicates were merged. Statistically significant FAIRE peaks were identified using CLC Genomics Workbench with input DNA as a control. Red bars indicate regions of FAIRE enrichment. The black rectangles highlight the RTA promoter.

To probe chromatin accessibility across the KSHV genome, we performed high throughput sequencing of FAIRE DNA (FAIRE-seq) isolated from DMSO and drug treated cells. We aligned the resulting sequencing reads to the KSHV reference genome (NC_009333) and used the CLC Genomics Workbench software to identify statistically significant FAIRE peaks. Consistent with previously published reports [22], the majority of the viral genome in DMSO treated cells was associated with nucleosomes (Fig 7A). As expected, FAIRE enrichment was detected at the RTA promoter in Romidepsin and Panobinostat treated samples. FAIRE peaks were identified across the KSHV genome in these samples, indicating that these compounds increase chromatin accessibility globally. In PTC-209 treated samples, FAIRE enrichment was detected at the RTA promoter compared to the DMSO control as expected (Fig 7B). However, treatment with PTC-209 did not induce dramatic changes in nucleosome occupancy across the KSHV genome.

### Silencing Bmi1 induces KSHV reactivation from latency

**Fig 8:**
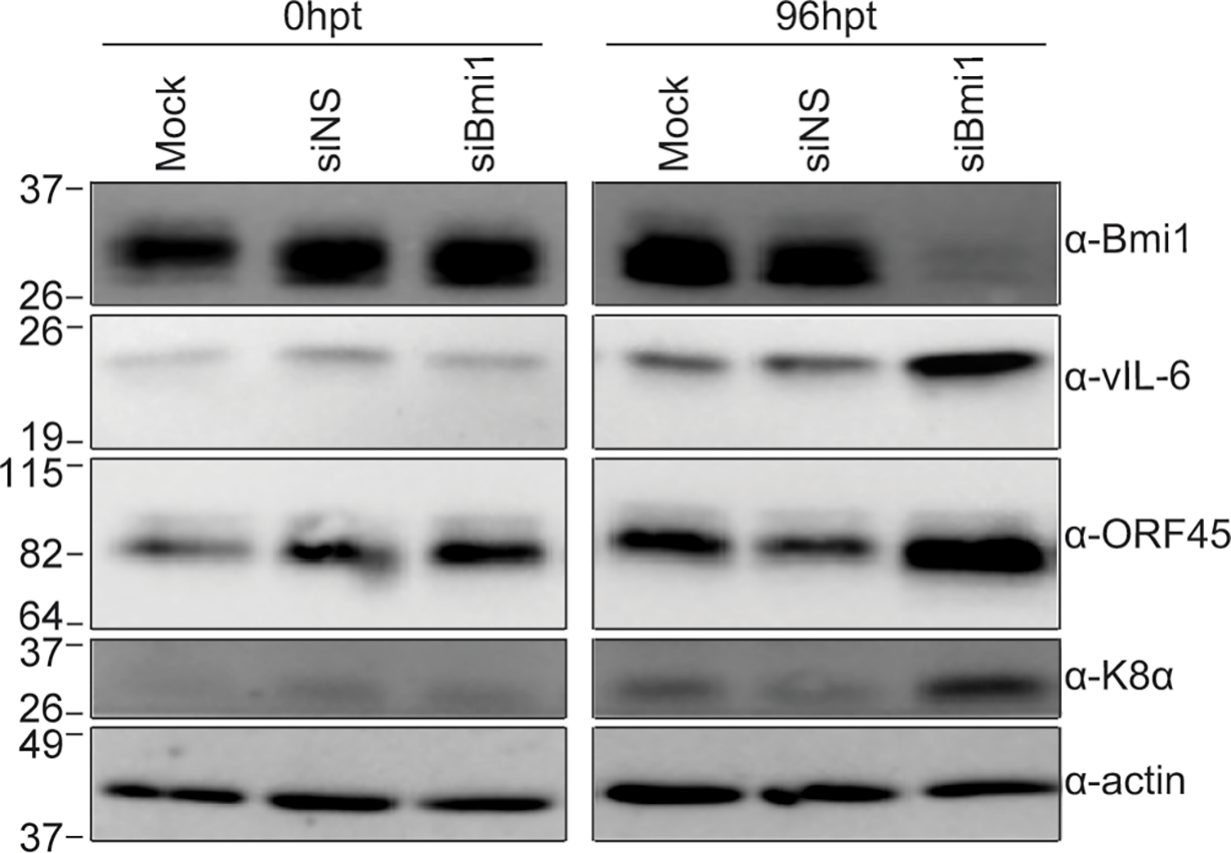
Bmi1 is essential for maintenance of KSHV latency. BCBL-1 cells were mock transfected or transfected with control or Bmi1 siRNAs, and cells were lysed at 0 and 96 hours post-transfection. KSHV lytic proteins were assayed by immunoblot.

To confirm a role for Bmi1 in maintaining KSHV latency, we silenced Bmi1 using siRNAs and performed immunoblot analysis for KSHV lytic proteins. BCBL-1 cells treated with Bmi1 siRNAs showed a marked decrease in Bmi1 expression compared to mock or control siRNA treated cells at 96 hours post-transfection (Fig 8). Bmi1 depletion resulted in increased levels of vIL-6, ORF45, and K8α. These data indicate that Bmi1 is critical for suppressing KSHV lytic gene expression.

## Discussion

In this study, we aimed to identify host chromatin-modifying proteins that are critical for the maintenance of KSHV latency. To this end, we screened a collection of inhibitors that target chromatin associated proteins, including histone writers, readers, and erasers, for their ability to stimulate KSHV reactivation. Romidepsin and Panobinostat, two HDAC inhibitors not previously shown to induce KSHV reactivation, as well as (+)-JQ1, a bromodomain inhibitor, and PTC-209, a Bmi1 inhibitor, induced KSHV lytic gene transcription. While Romidepsin and Panobinostat stimulated expression of all viral genes (Fig 3A), only a subset of viral genes were induced by (+)-JQ1 and PTC-209 (Fig 3B), suggesting that modulation of different epigenetic signals may activate viral gene transcription through different pathways. This possibility will be explored in future investigation. Romidepsin, Panobinostat, and PTC-209 induced viral DNA replication and the production of infectious virions. Histone modifications at the RTA promoter were altered by treatment with these compounds, where Romidepsin and Panobinostat increased H3 acetylation, and PTC-209 decreased H2AK119ub. Furthermore, each of these compounds induced nucleosome depletion at the RTA promoter. These data indicate that histone deacetylation and monoubiquitination at the RTA promoter aid KSHV latency, and conversely, that these marks need to be reversed to allow RTA transcription.

Silencing of PRC1 member Bmi1 by siRNA resulted in KSHV reactivation, demonstrating that PRC1 is critical for the maintenance of KSHV latency. Thus, it appears that PRC1 activity must be suppressed during reactivation. The upstream signals that govern activity of this complex, and how Bmi1 contributes to it in the context of KSHV infection, are not completely understood. The function of PRC1 components, including Bmi1, is modulated by post-translational modifications like SUMOylation and phosphorylation. For example, Bmi1 phosphorylation by MAPKAP kinase 3 results in dissociation of PRC1 complexes from chromatin [37, 38]. MAPKAP kinase 3 is downstream of activated ERK and p38, and ERK and p38 signaling have previously been implicated in KSHV reactivation [39–41]. Thus, the function of ERK or p38 signaling in KSHV reactivation may be to modulate PRC1 activity. Furthermore, LANA plays a role in PRC1 regulation [6, 42], which now can be further defined. The activity of the PRC1 complex may also be impacted by other histone modifications, as canonical PRC1 complexes contain chromobox proteins that bind H3K27me3. Therefore, a decrease in H3K27me3 at the RTA promoter may precede reduced PRC1 activity, resulting in KSHV reactivation. On the other hand, there is increasing evidence that non-canonical PRC1 complexes lacking chromobox proteins can catalyze H2AK119ub in the absence of H3K27me3 recognition [43, 44]. Clearly, a number of counteracting factors converge to establish the histone code on the RTA promoter. This study added Bmi1 as a new contributor, confirmed the preeminent role of bromodomain-containing proteins, and identified two novel, high-potency HDAC inhibitors to aid our understanding of this crucial point in the viral life cycle.

A thorough understanding of the factors controlling KSHV reactivation from latency is important for the development of therapies to combat KSHV-associated cancers or eradicate KSHV in HIV-positive patients. One approach that has been explored for KSHV as well as EBV, another gammaherpesvirus that causes lymphoproliferative disorders, is “lytic induction therapy,” which involves stimulation of reactivation in the presence of antiviral drugs. This approach has been shown to be effective in reducing lymphoproliferative disease and PEL growth in mouse xenograft models of pathogenesis [27, 45–47]. In the clinic, it relies as much on direct pan-cancer effects of HDAC inhibitors like SAHA (Vorinostat), as on KSHV-directed reactivation and virus-induced killing [48, 49]. Romidepsin is promising in this regard as it has previously been shown to be more effective than other HDAC inhibitors in stimulating reactivation of HIV in *ex vivo* patient samples [50] and has anti-lymphoma activity (reviewed in [51]). Furthermore, Bmi1 inhibition represents a new target for induction of KSHV reactivation from latency.

## Materials and methods

### Cell culture

BJAB-KSHV.219 cells (a kind gift from Thomas Schulz) [52] were maintained in RPMI media (Corning) supplemented with 10% FBS, 1% penicillin and streptomycin (Corning), 1% L-glutamine (Corning), and 4μg/mL puromycin (Corning). 293-KSHV.219 cells were previously described [24], and were maintained in DMEM media (Corning) supplemented with 10% FBS, 1% penicillin and streptomycin, 1% L-glutamine, and 1μg/mL puromycin. PEL cells were maintained in RPMI media supplemented with 10% FBS, 1% penicillin and streptomycin, 1% L-glutamine, 0.075% sodium bicarbonate (Corning), and 0.05mM β-mercaptoethanol (Sigma). PEL cells were reactivated with 1 mM NaB (Sigma) and 25 ng/mL TPA (Sigma) where indicated. Vero cells were maintained in DMEM media supplemented with 10% FBS, 1% penicillin and streptomycin, and 1% L-glutamine.

### Inhibitors

The compounds used in the epigenetic inhibitor screen are a custom set assembled by the UNC Center for Integrative Chemical Biology and Drug Discovery through purchase, synthesis, or from collaborators. More information about the source of each compound is available upon request. For a detailed list of the cellular targets of each compound included in the screen, see S1 Table. Romidepsin and Panobinostat were purchased from MedChemExpress. (+)-JQ1, PTC-209, and DZNep were purchased from Cayman Chemical.

### Inhibitor screen

4 x 10^4^ BJAB-KSHV.219 cells were seeded in a 96-well plate, or 1 x 10^5^ 293-KSHV.219 cells were seeded in a 48-well plate, and inhibitors were added the following day to a final concentration of 0.1% DMSO. After 48 or 120 hours of incubation, cells were fixed in 4% methanol-free formaldehyde (Polysciences, Inc) in PBS for 10 minutes at room temperature. Flow cytometry was performed using a Becton Dickinson LSRII, and analysis was performed using FlowJo software (Tree Star).

### RT-qPCR

Total RNA was isolated from cells using the RNeasy Plus Mini Kit (Qiagen), and cDNA synthesis was performed using the iSCRIPT cDNA Synthesis Kit (Bio-Rad) according to the manufacturer’s protocols. Primers used for SYBR green RT-qPCR are as follows:

Human GAPDH F: 5’- GGTGGTCTCCTCTGACTTCAACA; R: 5’-GTTGCTGTAGCCAAATTCGTTGT

KSHV vIL-6 F:5’- CGGTTCACTGCTGGTATCTG; R: 5’- CAGTATCGTTGATGGCTGGT

KSHV ORF57 F: 5’- TGGACATTATGAAGGGCATCCTA; R: 5’- CGGGTTCGGACAATTGCT

KSHV K8.1 F: 5’- AAAGCGTCCAGGCCACCACAGA; R: 5’- GGCAGAAAATGGCACACGGTTAC

The relative amount of vIL-6, ORF57, and K8.1 mRNA was normalized to GAPDH mRNA levels in each sample, and the fold difference between DMSO and compound treated samples was calculated.

### Western blot analysis

BCBL-1 cells were treated with DMSO, NaB+TPA, or 1μM Romidepsin or Panobinostat for 48 hours. BCBL-1 cells were treated with DMSO, NaB+TPA, or 10μM (+)-JQ1 or PTC-209 for 120 hours, and fresh compounds were added for an additional 48 hours. Cells were harvested, washed with PBS, and lysed in NP-40 lysis buffer (0.5% NP40, 150mM NaCl, 50mM Tris-HCL pH 8.0, 1x Complete Protease Inhibitor (Roche)). Protein concentration was determined by Bradford assay, and equivalent quantities of protein (15–30μg) were loaded per lane. Proteins were resolved by SDS-PAGE and transferred to a nitrocellulose membrane. The following primary antibodies were used: mouse anti-KSHV ORF45 (2D4A5; Thermo Scientific), mouse anti-K8α (8C12G10G1; Santa Cruz Biotechnology), rabbit anti-Bmi1 (D20B7; Cell Signaling), and goat anti-actin, horseradish peroxidase (HRP)-conjugated (C-11; Santa Cruz). Supernatants from ATCC hybridoma PTA-2220 (v6m 12.1.1) [53] were used to probe for vIL-6.

### KSHV array

To quantify KSHV mRNA levels, we used a real time qPCR array as previously described [54]. Briefly, 192 primer pairs targeting multiple sites near the 3’ end of each annotated KSHV open reading frame were used. Multiple reference genes for cellular transcripts were included for normalization. The array results in amplification reactions with similar efficiencies and annealing temperatures and thus allows us to directly compare the expression levels among different mRNAs. The qPCR arrays were plated in 384-well plates using the Tecan Freedom Evo liquid handling robot and cycled using Roche LightCycler 480. A detailed, step-by-step protocol is available at http://www.med.unc.edu/vironomics/protocols.

### Viral load assay

DNA was extracted from BCBL-1 cells treated with DMSO or 1 μM Romidepsin or Panobinostat for five days, or with DMSO or 10 μM PTC-209 for seven days. DNA was extracted from Vero cells 96 hours post infection with supernatants collected from drug treated BCBL-1 cells. DNA was isolated using the DNeasy Blood and Tissue Kit (Qiagen) following the manufacturer’s protocol. The ORF57 primers listed above were used to detect viral genomes, and genome copies were quantitated by comparison to an ORF57 plasmid-derived standard curve.

### KSHV infections

Supernatants were collected from BCBL-1 cells following treatment with DMSO or 1μM Romidepsin or Panobinostat for five days, or DMSO or 10μM PTC-209 for seven days. Naïve Vero cells were spinoculated with filtered supernatant at 2500rpm for 90min at 30°C. To determine KSHV genome copy number, cells were harvested 96 hours post-infection, and genome copies were determined by qPCR as described above. To assay viral gene expression, cells were harvested at 48 hours post-infection and RT-qPCR was performed as described above.

### ChIP-qPCR

For ChIP-qPCR analysis of H3Ac, BCBL-1 cells were treated with DMSO or 1μM Romidepsin or Panobinostat for 4 hours. For ChIP-qPCR analysis of H2AK119ub, BCBL-1 cells were treated with DMSO or 10μM PTC-209 for 120 hours. Cells were fixed with 1% (v/v) formaldehyde for 10 minutes at room temperature, and crosslinking was quenched with the addition of glycine (to 125mM) for five minutes at room temperature. Cells were washed with PBS and lysed in Cell Lysis Buffer (5mM Tris-HCl, pH 8.0, 85mM KCl, 0.5% NP-40, 1x cOmplete Protease Inhibitor (Roche)) for 10 minutes on ice. Lysates were centrifuged at 4000rpm for 5 minutes at 4°C, and nuclei were resuspended in RIPA buffer (150mM NaCl, 1.0% NP-40, 0.5% sodium deoxycholate, 0.1% SDS, 50mM Tris-HCl, pH 8.0, 1x cOmplete Protease Inhibitor) and sonicated to generate DNA fragments of ~300bp. Antibody coated beads were prepared by incubation of 2μg of each antibody with ChIP-Grade Protein A/G Plus Agarose (Thermo Scientific) for 1 hour at room temperature, followed by overnight blocking with 5mg/mL BSA at 4°C. The following antibodies were used: normal rabbit IgG (Millipore), rabbit anti-acetyl-H3 (06–599; Millipore), rabbit anti-H3 (ab1791; Abcam), rabbit anti-H2AK119ub (D27C4; Cell Signaling), rabbit anti-H2A (D603A; Cell Signaling). DNA was pre-cleared by incubation with agarose for one hour at 4°C, and 1x10^7^ nuclei were added to antibody-bound agarose and incubated overnight at 4°C. Beads were washed 2X with RIPA, 2X with Low Salt Buffer (0.1% SDS, 1% NP-40, 2mM EDTA, 20mM Tris-HCl, pH 8.0, 150mM NaCl), 2X with High Salt Buffer (0.1% SDS, 1% NP-40, 2mM EDTA, 20mM Tris-HCl pH 8.0, 500mM NaCl), 2X with LiCl Buffer (0.25M LiCl, 1% NP-40, 1% sodium deoxycholate, 1mM EDTA, 20mM Tris-HCl, pH 8.0), and 2X with TE. DNA was eluted with Elution Buffer (100mM NaHCO3, 1% SDS) for 30min at 65°C, and crosslinks were reversed with De-crosslinking Buffer (500mM NaCl, 2mM EDTA, 20mM Tris-HCl, pH 8.0, 0.5mg/mL Proteinase K) overnight at 65°C. DNA was purified with the Qiagen PCR Purification Kit following the manufacturer’s protocol. qPCR of the RTA promoter was performed with the following primers:

KSHV RTA promoter Region 1 F: 5’- CAAAGGGGTATTGCTCCGGT; R: 5’- CTCCTTGGTGGGTCAGTGTC

KSHV RTA promoter Region 2 F: 5’- CCGCCATACTCTTCCAGGAC; R: 5’- AGCACATTACCTCGGACGTG

KSHV RTA promoter Region 3 F: 5’- CACCAGGGACGCTAAGAACC; R: 5’- GGTACCACATCGGGTTTCGT

KSHV RTA promoter Region 4 F: 5’- CCCCATAGGACCCAGCTACA; R: 5’- GGCTTAATGAGTCGCCGGTA

### FAIRE-qPCR

1x10^7^ 293-KSHV.219 cells were treated with DMSO or 1 μM Romidepsin or Panobinostat for 48 hours, or DMSO or 10 μM PTC-209 for 120 hours. Cells were crosslinked, quenched, lysed, and homogenized using the ChIP-IT High Sensitivity Chromatin Preparation Kit (Active Motif) according to the manufacturer’s protocol. Nuclei were resuspended in 100μL FAIRE Lysis Buffer A (10mM Tris-HCl, pH 8.0, 2% Triton X-100, 1% SDS, 100mM NaCl, 1mM EDTA) and sonicated to generate DNA fragments of ~300bp. DNA was treated with 1μg/μL RNase A for 30 minutes at 37°C. “Input” DNA was prepared from 10% of the sample by incubation with 0.2μg/μL Proteinase K for 1 hour at 55°C followed by overnight incubation at 65°C. Input and FAIRE DNA were purified using the ChIP DNA Clean & Concentrator Kit (Zymo Research). The same primers used for ChIP-qPCR of the RTA promoter were used here. The AURKAIP1 primers are as follows:

Human AURKAIP1 F: 5’- TATACCCGCAGGTCCAGAATCGTT; R: 5’- AATAGCTCTAGACGCTTCCGCCTT

### KSHV FAIRE-seq

Input and FAIRE DNA library preparation and sequencing was performed by Genewiz, Inc on an Illumina HiSeq. Reads were imported into CLC Genomics Workbench and trimmed using a quality limit of 0.05. Trimmed reads were mapped to the KSHV reference genome NC_009333 with default mismatch rates applied and length and similarity fractions at 90%. Mapped reads from biological replicates were merged, and the CLC Genomics Workbench “ChIP-Seq Analysis” tool was used for peak calling using the input DNA as a control and a P value cutoff of 5x10^-4^ for the Romidepsin and Panobinostat treated samples, and 5x10^-3^ for the PTC-209 treated samples.

### siRNA transfection

The following chemically synthesized siRNAs were obtained from Dharmacon GE: ON-TARGETplus Non-targeting Control siRNAs #1 (D-001810-01); ON_TARGETplus Human Bmi1 siRNA SMARTpool (L-005230-01). BCBL-1 cells were transfected using the Amaxa Cell Line Nucleofector Kit V (Lonza) according to the manufacturer’s protocol, and cells were lysed at 0, 48, and 96 hours post-transfection in NP-40 lysis buffer for Western blot analysis.

## Supporting information

S1 Fig Inhibitor screen for epigenetic regulatory proteins that induce KSHV reactivation from latency in 293-KSHV.219 cells. 293-KSHV.219 cells were incubated with 1 μM of each compound for 48 hours in (A) or 10 μM of each compound for 120 hours in (B), and the percentage of GFP positive cells expressing RFP (percent reactivation) was determined by FACS analysis. Data are representative of two independent experiments performed in duplicate; mean and standard error are shown.
(TIF)

S1 Table List of compounds included in epigenetic inhibitor screen and their cellular targets. (DOCX)
