# Supplementary figures and images for: Chromatin remodeling controls Kaposi's sarcoma-associated herpesvirus reactivation from latency
Source: PLoS Pathog. 2018 Sep 13;14(9):e1007267. doi: 10.1371/journal.ppat.1007267 (PMC6136816; doi:10.1371/journal.ppat.1007267)

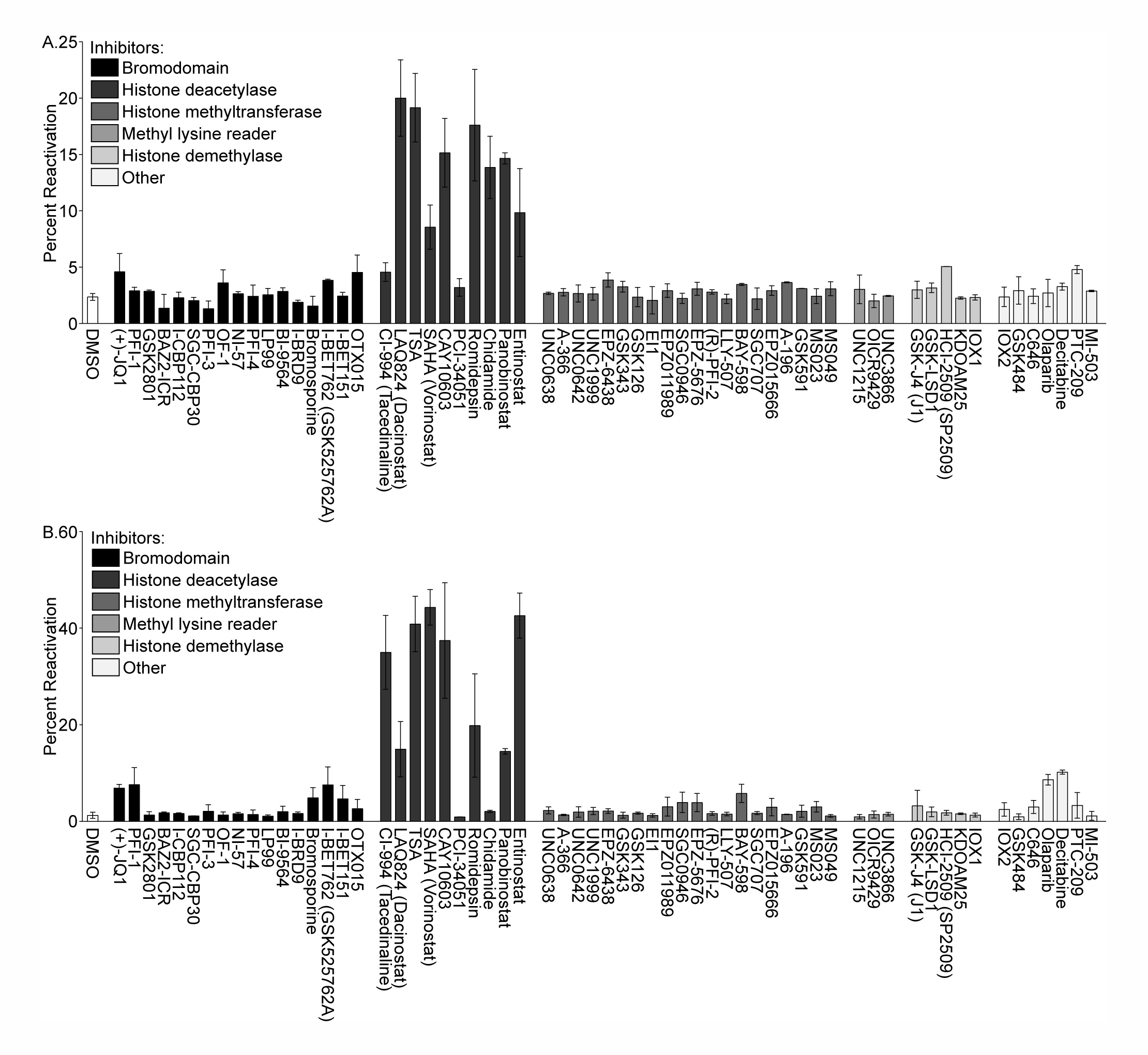

Supplement: S1 Fig — 293-KSHV.219 cells were incubated with 1 μM of each compound for 48 hours in (A) or 10 μM of each compound for 120 hours in (B), and the percentage of GFP positive cells expressing RFP (percent reactivation) was determined by FACS analysis. Data are representative of two independent experiments performed in duplicate; mean and standard error are shown. (TIF) [file ppat.1007267.s001.tif]
