# Supplementary material for: Chromatin remodeling controls Kaposi's sarcoma-associated herpesvirus reactivation from latency
Source: PLoS Pathog. 2018 Sep 13;14(9):e1007267. doi: 10.1371/journal.ppat.1007267 (PMC6136816; doi:10.1371/journal.ppat.1007267)
Supplement: S1 Table — (DOCX) [file ppat.1007267.s002.docx]

**Supplementary Table 1. List of compounds included in epigenetic inhibitor screen and their cellular targets.**

| **Compound Name** | **Protein Target(s)*** | **Target Class** |
| --- | --- | --- |
|  |  |  |
| (+)-JQ1 | BET family | bromodomain |
| PFI-1 | BET family | bromodomain |
| GSK2801 | BAZ2A/B | bromodomain |
| BAZ2-ICR | BAZ2A/B | bromodomain |
| I-CBP112 | CREBBP/EP300 | bromodomain |
| SGC-CBP30 | CREBBP/EP300 | bromodomain |
| PFI-3 | SMARCA2/4, PBI(5) | bromodomain |
| OF-1 | BRPF family | bromodomain |
| NI-57 | BRPF family | bromodomain |
| PFI-4 | BRPF1 | bromodomain |
| LP99 | BRD9/7 | bromodomain |
| BI-9564 | BRD9/7 | bromodomain |
| I-BRD9 | BRD9 | bromodomain |
| Bromosporine | bromodomains | bromodomain |
| I-BET762 (GSK525762A) | BET family | bromodomain |
| I-BET151 | BET family | bromodomain |
| OTX015 | BET family | bromodomain |
| Cl-994 (Tacedinaline) | HDAC1,2,3 | HDAC |
| LAQ824 (Dacinostat) | HDACs | HDAC |
| TSA | HDACs (except 8) | HDAC |
| SAHA (Vorinostat) | HDAC1,2,3,6 | HDAC |
| CAY10603 | HDAC6 | HDAC |
| PCI-34051 | HDAC8 | HDAC |
| Romidepsin | HDACs | HDAC |
| Chidamide | HDACs | HDAC |
| Panobinostat | HDACs | HDAC |
| Entinostat | HDAC1,3 | HDAC |
| UNC0638 | G9a/GLP | HMT |
| A-366 | G9a/GLP | HMT |
| UNC0642 | G9a/GLP | HMT |
| UNC1999 | EZH2/EZH1 | HMT |
| EPZ-6438 | EZH2 | HMT |
| GSK343 | EZH2 | HMT |
| GSK126 | EZH2 | HMT |
| EI1 | EZH2 | HMT |
| EPZ011989 | EZH2 | HMT |
| SGC0946 | DOT1L | HMT |
| EPZ-5676 | DOT1L | HMT |
| (R)-PFI-2 | SETD7 | HMT |
| LLY-507 | SMYD2 | HMT |
| BAY-598 | SMYD2 | HMT |
| SGC707 | PRMT3 | HMT |
| EPZ015666 | PRMT5 | HMT |
| A-196 | SUV420H1/H2 | HMT |
| GSK591 | PRMT5 | HMT |
| MS023 | Type I PRMT | HMT |
| MS049 | PRMT4/6 | HMT |
| UNC1215 | L3MBTL3 | Kme Reader |
| OICR9429 | WDR5 | Kme Reader |
| UNC3866 | CBX4/7 | Kme Reader |
| GSK-J4 (J1) | JMJD3/UTX | Demethylase |
| GSK-LSD1 | LSD1 | Demethylase |
| HCl-2509 (SP2509) | LSD1 | Demethylase |
| KDOAM25 | JARID1/KDM5 | Demethylase |
| IOX1 | 2-OG | Demethylase |
| IOX2 | Prolyl hydroxylase | Other |
| GSK484 | PAD-4 | Other |
| C646 | p300 (HAT domain) | Other |
| Olaparib | PARP | Other |
| Decitabine | DNMTs | Other |
| PTC-209 | BMI-1 | Other |
| MI-503 | Menin-MLL | Other |

*The primary targets reported are listed; however, in some cases the compounds have off-target activities which are not included here.
